# Application of machine learning in the prevention of periprosthetic joint infection following total knee arthroplasty: a systematic review

**DOI:** 10.1186/s42836-023-00195-2

**Published:** 2023-06-14

**Authors:** Yuk Yee Chong, Ping Keung Chan, Vincent Wai Kwan Chan, Amy Cheung, Michelle Hilda Luk, Man Hong Cheung, Henry Fu, Kwong Yuen Chiu

**Affiliations:** 1 Department of Orthopaedics and Traumatology, School of Clinical Medicine, The University of Hong Kong, Hong Kong SAR, China; 2 Department of Orthopaedics and Traumatology, Queen Mary Hospital, Hong Kong SAR, China

**Keywords:** Artificial intelligence (AI), Machine learning, Deep learning, Periprosthetic joint infection (PJI), Surgical site infection (SSI), Total knee arthroplasty (TKA), Replacement

## Abstract

**Background:**

Machine learning is a promising and powerful technology with increasing use in orthopedics. Periprosthetic joint infection following total knee arthroplasty results in increased morbidity and mortality. This systematic review investigated the use of machine learning in preventing periprosthetic joint infection.

**Methods:**

A systematic review was conducted according to the Preferred Reporting Items for Systematic Reviews and Meta-Analyses guidelines. PubMed was searched in November 2022. All studies that investigated the clinical applications of machine learning in the prevention of periprosthetic joint infection following total knee arthroplasty were included. Non-English studies, studies with no full text available, studies focusing on non-clinical applications of machine learning, reviews and meta-analyses were excluded. For each included study, its characteristics, machine learning applications, algorithms, statistical performances, strengths and limitations were summarized. Limitations of the current machine learning applications and the studies, including their ‘black box’ nature, overfitting, the requirement of a large dataset, the lack of external validation, and their retrospective nature were identified.

**Results:**

Eleven studies were included in the final analysis. Machine learning applications in the prevention of periprosthetic joint infection were divided into four categories: prediction, diagnosis, antibiotic application and prognosis.

**Conclusion:**

Machine learning may be a favorable alternative to manual methods in the prevention of periprosthetic joint infection following total knee arthroplasty. It aids in preoperative health optimization, preoperative surgical planning, the early diagnosis of infection, the early application of suitable antibiotics, and the prediction of clinical outcomes. Future research is warranted to resolve the current limitations and bring machine learning into clinical settings.

## Background

Total knee arthroplasty (TKA) is a common procedure for severe knee osteoarthritis and other end-stage joint conditions. One complication of TKA is periprosthetic joint infection (PJI). The incidence of PJI ranges from 1% to 2% following primary TKA [1]. This complication results in an increased cost of treatments, a prolonged hospital stay, an increase in pain, and an increase in morbidity and mortality [2, 3]. Currently, machine learning (ML) is becoming a promising and powerful technology in the prevention of PJI as it may benefit the prediction, diagnosis, treatment and prognosis of PJI.

**Fig. 1:**
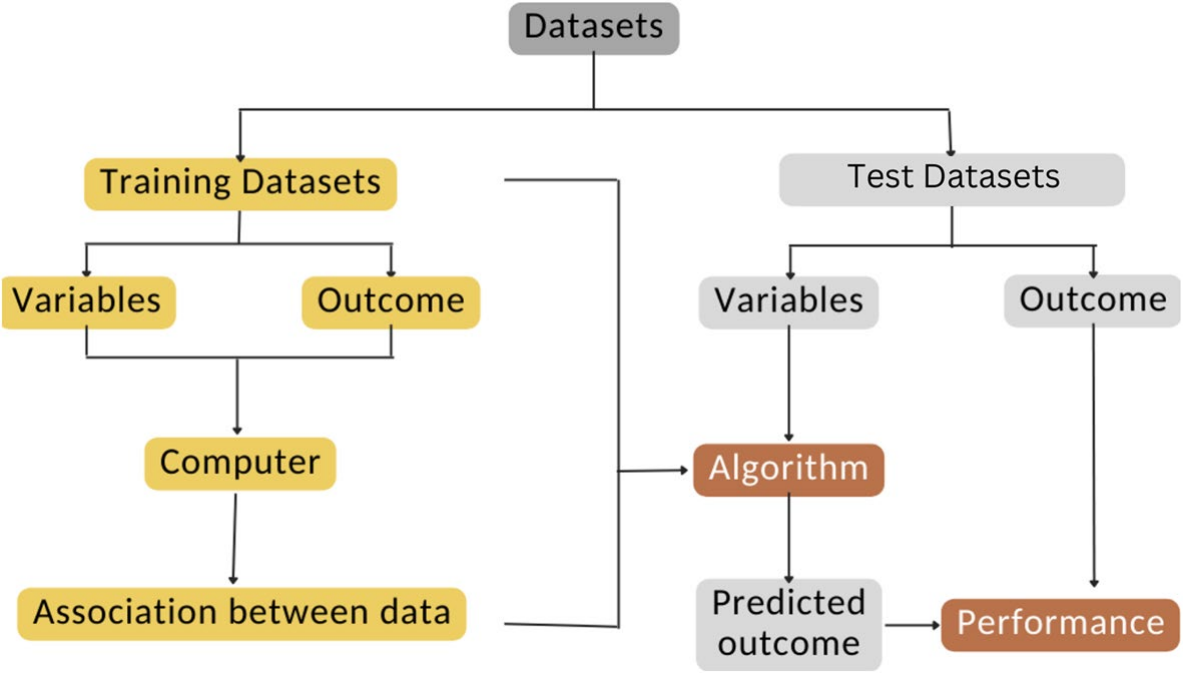
Process of ML development

ML is a form of artificial intelligence. It may imitate human thinking and may even exceed human capability [4]. To build a ML model, massive datasets and outcomes could be split into a training set and a test set, and input into a computer (Fig. 1) [5]. Then, the computer may find an association between the data and generate an algorithm accordingly. Various hyperparameters could be adjusted to improve the algorithm’s performance [6]. The final algorithm could be used to generate decisions in future unseen datasets [5].

ML is a promising field with surging applications. Some ML models have been developed for the prevention of PJI. A complete prevention strategy for PJI usually includes four key principles, i.e., early prediction, diagnosis, antibiotic application, and prognosis. The ML models built by Yeo et al. [7] and Kuo et al. [8] enabled early prediction and diagnosis of PJI respectively, allowing for patient-specific surgical planning and detection of infection. Luftinger et al. [9] reviewed multiple ML models for determining the antibiotic susceptibility status of common PJI pathogens, allowing for an early prescription of antibiotics for PJI management. Wouthuyzen et al. [10] reviewed an ML model that could predict outcomes more accurately than two statistical risk scores for debridement, antibiotics and implant retention (DAIR). One of the common drawbacks of the models was the difficulty in interpreting the results. Although a few studies investigated the use of ML in the prevention of PJI, to our best knowledge, so far, no systematic reviews on the prevention of PJI covered all four aforementioned key principles.

This systematic review investigated the use of ML designed for prophylaxis of PJI. We also elaborated on and summarized the efficiency of the prevention strategy based on the four key principles, i.e., early prediction, diagnosis, antibiotic application, and prognosis.

## Materials and methods

### Search and selection

A systematic review of the literature published from 2006 to 2022 was conducted based on the Preferred Reporting Items for Systematic Reviews and Meta-Analyses guidelines [11]. PubMed was searched in November 2022 using the following keywords: ‘periprosthetic joint infection’, ‘prosthetic joint infection’, ‘PJI’, ‘infection’, ‘artificial intelligence’, ‘AI’, ‘machine learning’, ‘ML’, ‘deep learning’, ‘joint replacement’ and ‘arthroplasty’. References of eligible studies were included in the search for additional results.

Two independent reviewers reviewed the studies. Discrepancies between the reviewers were resolved by comparing notes. All studies that examined the clinical applications of ML in the prevention of PJI following TKA were included. Non-English studies, studies with no full text available, studies focusing on non-clinical applications of ML, reviews and meta-analyses were excluded. The retrieved studies were first screened for possible relevance to the review topic by reading the titles and abstracts. Then, full texts were perused to further confirm eligibility.

### Quality assessment

Two independent reviewers assessed the methodological quality by employing the National Institutes of Health quality assessment tool for case-control studies [12]. Discrepancies in the quality rating were resolved through discussion. The total quality score was calculated as the number of ‘yes’ over the number of questions, with questions answered with ‘not applicable’ excluded. The quality was rated as good (>75%), fair (50%–75%) or poor (<50%). The total score and quality rating are listed in Table 1.

**Table 1:** Quality of the 11 studies included

**First author [Ref.]**	**Year**	**Total score**	**Quality rating**
Yeo, I. [7]	2022	6/11 (54.5%)	Fair
Kuo, F.C. [8]	2021	6/11 (54.5%)	Fair
Tao, Y. [13]	2022	6/11 (54.5%)	Fair
Davis, J.J. [14]	2016	8/11 (72.7%)	Fair
Drouin, A. [15]	2019	7/11 (63.6%)	Fair
Moradigaravand, D. [16]	2018	8/11 (72.7%)	Fair
Nguyen, M. [17]	2018	7/11 (63.6%)	Fair
Khaledi, A. [18]	2020	7/11 (63.6%)	Fair
Aun, E. [19]	2018	7/11 (63.6%)	Fair
Shohat, N. [20]	2020	6/11 (54.5%)	Fair
Klemt, C. [21]	2021	6/11 (54.5%)	Fair

### Data extraction

Data extracted from each study consisted of three parts: (1) the characteristics of the studies, including the first author, the title, the journal of publication, the year of publication and the cohort size, (2) the details of the ML models, including their applications, algorithms and statistical performances and (3) the strengths and limitations of the studies.

## Results

**Fig. 2:**
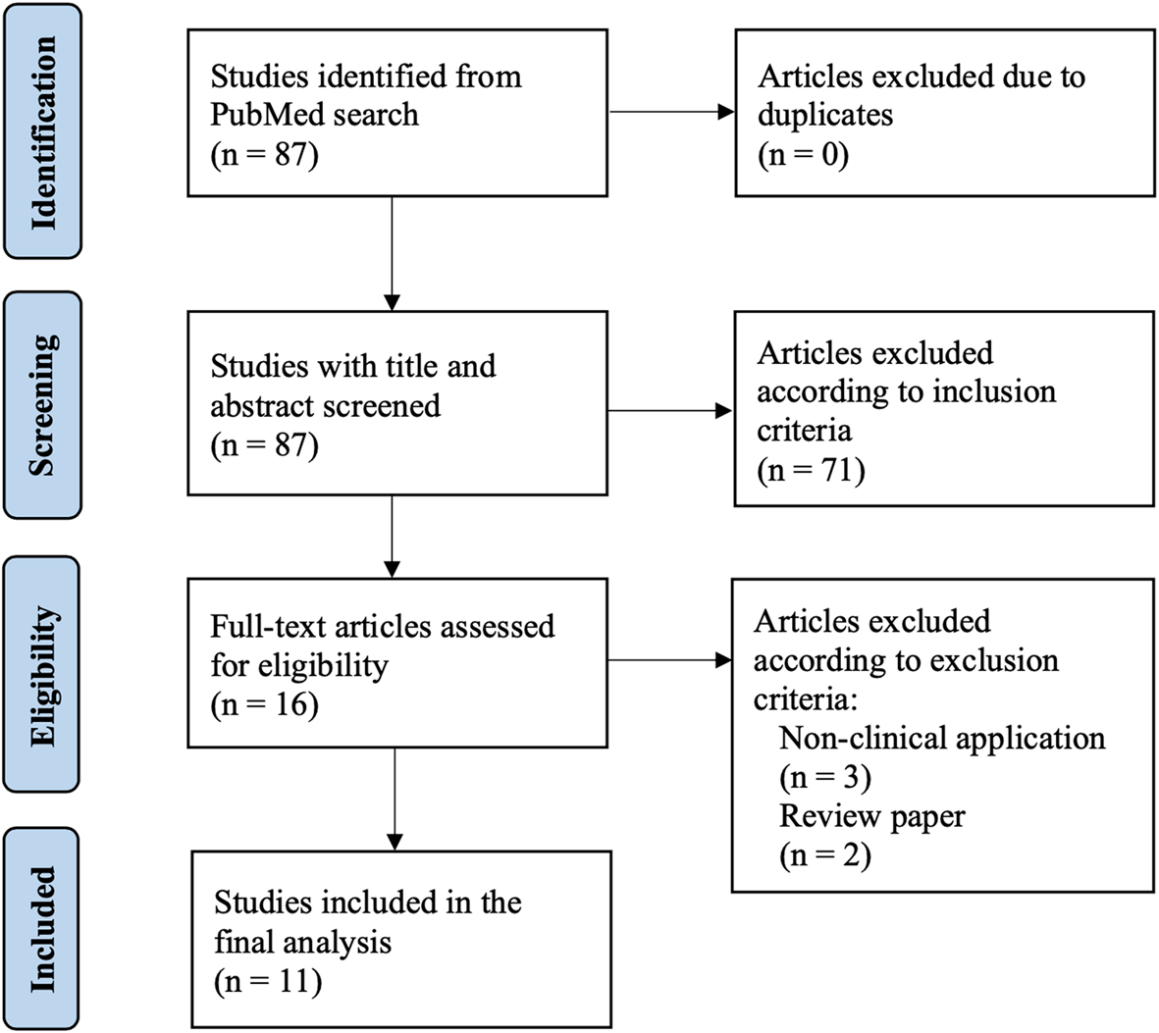
Preferred Reporting Items for Systematic Reviews and Meta-Analyses flowchart of our review process

**Fig. 3:**
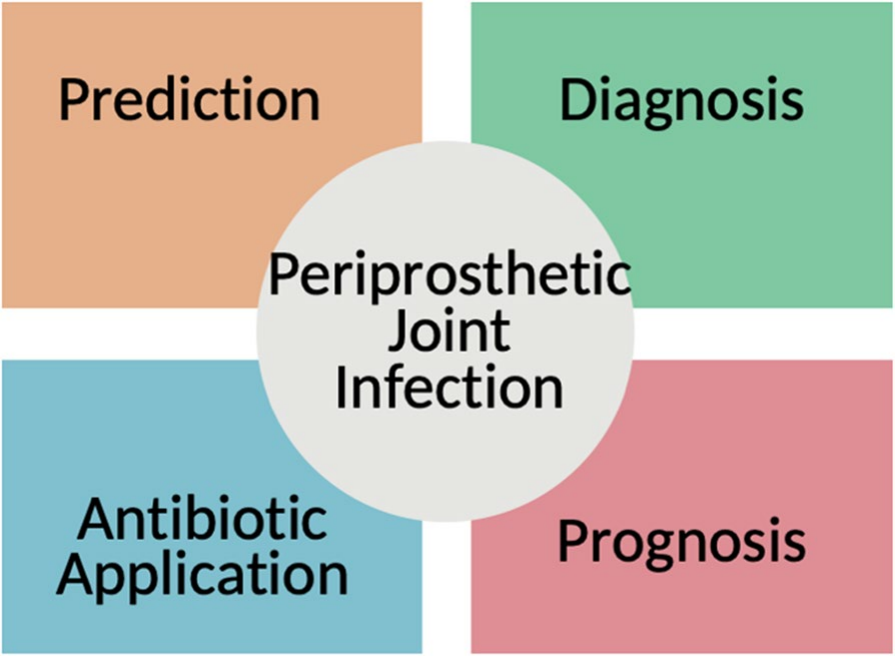
Four areas of ML application in the prevention of PJI

The initial search identified 87 studies (Fig. 2). Seventy-one studies were excluded after reading the titles and abstracts against the inclusion and exclusion criteria. Full texts were obtained and reviewed for the remaining 16 studies. Five studies were removed, with 11 studies included for the final analysis [7, 8, 13–21]. The included studies targeted four areas of ML application: PJI prediction, diagnosis, antibiotic application, and prognosis (Fig. 3). The features of the studies are summarized in Table 2, in terms of PJI prediction, diagnosis and prognosis, and in Table 3 in terms of pathogens. The details of the ML models, strengths and limitations of the studies are summarized in Table 4 for PJI prediction, Table 5 for PJI diagnosis, Tables 6 and 7 for PJI pathogens, and Table 8 for PJI prognosis. The two most common metrics used by the included studies to evaluate the performance of the ML models in the classification of individuals was the area under the receiver operating characteristic curve (AUC) and accuracy. The receiver operating characteristics curve is a probability curve plotted with true positive rates against false positive rates. The AUC represents the degree of classification ability. The value of AUC ranges from 0 (poor model performance) to 1 (perfect model performance). The value of accuracy ranges from 0% (poor performance) to 100% (perfect performance). Other common metrics used were the Brier score, F1 score, sensitivity, specificity, calibration intercept and predictive value.

**Table 2:** Characteristics of five studies on prediction, diagnosis and prognosis of periprosthetic joint infection

**First author [Ref.]**	**Articles**	**Journal**	**Year**	**Number**
**Prediction**				
Yeo, I. [7]	The use of artificial neural networks for the prediction of surgical site infection following total knee arthroplasty	*The Journal of Knee Surgery*	2022	10021 patients
**Diagnosis**				
Kuo, F.C. [8]	Periprosthetic joint infection prediction via machine learning: comprehensible personalized decision support for diagnosis	*The Journal of Arthroplasty*	2021	323 patients
Tao, Y. [13]	A preliminary study on the application of deep learning methods based on convolutional network to the pathological diagnosis of periprosthetic joint infection	*Arthroplasty*	2022	20 patients (Training sets: 461 positive images, 461 negative images)
**Prognosis**				
Shohat, N. [20]	2020 Frank Stinchfield Award: identifying who will fail following irrigation and debridement for prosthetic joint infection	*The Bone & Joint Journal*	2020	609 patients
Klemt, C. [21]	Machine learning models accurately predict recurrent infection following revision total knee arthroplasty for periprosthetic joint infection	*Knee Surgery, Sports Traumatology, Arthroscopy*	2021	618 patients

**Table 3:** Characteristics of six studies on the pathogens of periprosthetic joint infection

**First author [Ref.]**	**Articles**	**Journal**	**Year**	**Number and pathogens isolated**
Davis, J.J. [14]	Antimicrobial resistance prediction in pathosystems resource integration center and rapid annotation using subsystem technology	*Scientific Report*	2016	606 *Staphylococcus aureus*
Drouin, A. [15]	Interpretable genotype-to-phenotype classifiers with performance guarantees	*Scientific Report*	2019	1593 *Staphylococcus aureus*, 134 *Enterococcus faecium*, 1524 *Escherichia coli*, 2107 *Klebsiella pneumoniae*, 491 *Pseudomonas aeruginosa*
Moradigaravand, D. [16]	Prediction of antibiotic resistance in *Escherichia coli* from large-scale pan-genome data	*PLoS Computational Biology*	2018	1936 *Escherichia coli*
Nguyen, M. [17]	Developing an in silico minimum inhibitory concentration panel test for *Klebsiella pneumoniae*	*Scientific Report*	2018	1668 *Klebsiella pneumoniae*
Khaledi, A. [18]	Predicting antimicrobial resistance in *Pseudomonas aeruginosa* with machine learning-enabled molecular diagnostics	*EMBO Molecular Medicine*	2020	414 *Pseudomonas aeruginosa*
Aun, E. [19]	A k-mer-based method for the identification of phenotype-associated genomic biomarkers and predicting phenotypes of sequenced bacteria	*PLoS Computational Biology*	2018	200 *Pseudomonas aeruginosa*

**Table 4:** A summary on the details of machine learning, strengths and limitations of the study on prediction of periprosthetic joint infection

**First author [Ref.]**	**Machine learning application**	**Algorithm**	**Statistical performance**	**Strengths**	**Limitations**
Yeo, I. (2022) [7]	Preoperative prediction of the risk of periprosthetic joint infection following primary total knee arthroplasty	Artificial neural network	Area under the receiver operating characteristic curve: 0.84. Accuracy: >94.0%. Brier score: 0.054. Calibration intercept: 0.09. Calibration slope: 1.06	Important variables for the risk prediction were identified and expressed in a bar graph which showed the relative importance value	No external validation

**Table 5:** A summary on the details of ML, strengths and limitations of the studies on diagnosis of PJI

**First author [Ref.]**	**ML application**	**Algorithm**	**Statistical performance**	**Strengths**	**Limitations**
Kuo, F.C. (2021) [8]	A personalized PJI diagnosis	Two-level stacked generalization architecture: -Meta-classifier: Support vector machine; -Base classifiers: Randomforest, eXtreme gradient boosting, logistic regression, naïve bayesian	AUC: 0.988. Accuracy: 96.4%. Recall: 0.981. F1 score: 0.97. Matthews correlation -coefficient: 0.926. Precision: 0.96	The performance outperformed that of International Consensus Meeting criteria; if-then rule was used for the explanation of the results	No external validation; small cohort size
Tao, Y. (2022) [13]	PJI pathological diagnosis	ResNet34 deep learning convolutional network	AUC: 0.8136. Average accuracy: 93.3%. Average recall rate: 0.9739. F1 score: 0.9482	External validation was conducted	Small cohort size

*AUC* Area under the receiver operating characteristic curve, *ML* Machine learning, *PJI* Periprosthetic joint infection

**Table 6:** A summary on the details of ML, strengths and limitations of the studies on pathogens of periprosthetic joint infection

**First author [Ref.]**	**ML application**	**Algorithm**	**Statistical performance**	**Strengths**	**Limitations**
Davis, J.J. (2016) [14]	Used ML-based NGS-based pAST method to predict the antibiotic susceptibility status of *Staphylococcus aureus* on methicillin	Adaptive boosting	Area under the receiver operating characteristic curve: 0.991. Accuracy: 99.5%. F1 score: 0.995	_	No external validation
Drouin, A. (2019) [15]	Used ML-based NGS-based pAST method to predict the antibiotic susceptibility status of: *Staphylococcus aureus*: Methicillin, *Enterococcus faecium*: Vancomycin, *Escherichia coli*: Amoxicillin/Clavulanic acid, *Klebsiella pneumoniae*: Gentamicin, *Pseudomonas aeruginosa*: Levofloxacin	Set Covering Machine	Accuracy: *Staphylococcus aureus*: 98.7%; *Enterococcus faecium*: 100.0%; *Escherichia coli*: 81.8%; *Klebsiella pneumoniae*: 95.0%; *Pseudomonas aeruginosa*: 93.9%	No prior knowledge of the genome was needed; some comprehensive tutorials were provided for visualization and annotation of the model	No external validation
Moradigaravand, D. (2018) [16]	Used ML-based NGS-based pAST method to predict the antibiotic susceptibility status of *Escherichia coli* to 11 antibiotics	Gradient-boosting decision tree	Average accuracy: 91.0% (range: 81.0%–97.0%)	The ML model’s performance was compared with a rule-based model; ML outperformed the rule-based model; no prior knowledge of the biological mechanism was needed	No external validation

*ML* Machine learning, *NGS* Next-generation sequencing, *PAST* Predictive antimicrobial susceptibility testing

**Table 7:** A summary on the details of ML, strengths and limitations of the studies on pathogens of periprosthetic joint infection

**First author [Ref.]**	**ML application**	**Algorithm**	**Statistical performance**	**Strengths**	**Limitations**
Nguyen, M. (2018) [17]	Used ML-based NGS-based pAST method to predict the MICs for *Klebsiella pneumoniae* in order to determine the degree of resistance status on 20 antibiotics	Gradient-boosting tree	Average accuracy: 92.0%	No prior knowledge of the gene content was needed; the continuous phenotype (MIC) was predicted	No external validation
Khaledi, A. (2020) [18]	Used ML-based NGS-based pAST method to predict the antibiotic susceptibility of *Pseudomonas aeruginosa* to 4 antibiotics (ceftazidime, meropenem, ciprofloxacin and tobramycin)	Support vector machine	-Using single-nucleotide polymorphism’*s* information or gene expression data alone: Sensitivity and Predictive value: 0.8–0.9 -In combination: Sensitivity and Predictive value: >0.9	No prior knowledge of molecular mechanisms of the resistance was needed	No external validation
Aun, E. (2018) [19]	Used ML-based NGS-based pAST method to predict the antibiotic susceptibility of *Pseudomonas aeruginosa* to ciprofloxacin	Logistic regression for binary phenotypes (susceptible or resistant); Linear regression for the continuous phenotype (MIC value)	-Logistic regression: Accuracy: 88.0%. F1-measure: 0.88. Sensitivity: 0.90. Specificity: 0.87 -Linear regression: Coefficient of determination (*R*^2^): 0.42. Pearson correlation coefficient: 0.68. Spearman correlation coefficient: 0.84	A simple software ‘PhenotypeSeeker’ was developed; the continuous phenotype (MIC) was predicted	No external validation

*MIC* Minimum inhibitory concentration, *ML* Machine learning, *NGS* Next-generation sequencing, *PAST* Predictive antimicrobial susceptibility testing

**Table 8:** A summary on the details of ML, strengths and limitations of the studies on prognosis of periprosthetic joint infection

**First author [Ref.]**	**ML application**	**Algorithm**	**Statistical performance**	**Strengths**	**Limitations**
Shohat, N. (2020) [20]	Preoperative prediction of the risk of debridement, antibiotics and implant retention failure	Random forest analysis	Area under the receiver operating characteristic curve: 0.74	Important variables for the risk prediction were identified and expressed in a bar graph showing the relative importance value	No external validation
Klemt, C. (2021) [21]	Preoperative prediction of the risk of recurrent periprosthetic joint infection following revision total knee arthroplasty	Artificial neural network	Area under the receiver operating characteristic curve: 0.84. Brier score: 0.053. Calibration intercept: 0.06. Calibration slope: 1.09	Important variables for the risk prediction were identified and expressed in a bar graph showing the importance value	No external validation

*ML* Machine learning

### Prediction

Yeo et al. [7] used an artificial neural network to develop an ML model for the preoperative risk prediction of both superficial surgical site infection and PJI following TKA. About 10,000 primary TKA patients were included. The average follow-up time lasted for about 3 years. The patients’ demographic and operational variables were collected. The model performance was good, with an AUC of 0.84 and a Brier score of 0.054 (a Brier score close to zero indicates good accuracy of probabilistic prediction). Several important variables for prediction were identified, including Charlson comorbidity score, obesity, smoking and diabetes.

### Diagnosis

Kuo et al. [8] developed a diagnostic model using a two-level stacked generalization architecture with a support vector machine as a meta-classifier. A small cohort of 323 patients was included. The model performance in diagnosing chronic PJI was compared with that of the 2018 European Bone and Joint Infection Society criteria. They applied an if-then rule and a decision diagram to visualize the decision pathway of the ML model. With an AUC of 0.988, the model performed better than the criteria of the International Consensus Meeting (ICM) with an AUC of 0.958. The accuracy of the model was 96.4%. The model not only identified most of the common important features listed in the 2018 ICM criteria for PJI diagnosis but also considered additional important features, such as hemoglobin and prothrombin time, and set up different baseline values for these features that were individualized to each patient.

An ML model based solely on pathological information is also available for diagnosing PJI. Using pathological data, Tao et al. [13] trained a resNet34 deep-learning convolutional network model to diagnose PJI. The cohort comprised 20 revision total knee and hip arthroplasty patients from the Chinese People’s Liberation Army General Hospital, who were classified into infected and non-infected based on the 2018 ICM guidelines. Frozen pathological sections collected were converted into electronic images with 461 positive and 461 negative images for model training.

Comparing the performance of the different ML models for PJI diagnosis, Kuo’s [8] model, which utilized a wide variety of demographic, biomedical, comorbidity, surgical and ICM-related data, demonstrated a greater performance in PJI diagnosis, yielding an AUC of 0.988 and an accuracy of 96.4%. This was followed by Tao’s [13] model with an AUC of 0.814 and an average accuracy of 93.3%. This might be due to a narrower and more specialized spectrum of data used by Tao’s model as compared to Kuo’s. Nevertheless, both of them provide a new research direction for PJI diagnosis.

### Antibiotic application

#### *Staphylococcus aureus*

Davis et al. [14] developed an adaptive boosting ML classifier for the prediction of methicillin resistance status in *Staphylococcus aureus*. A total of 606 bacterial genomes were collected from the Pathosystems Resource Integration Center database. The DNA k-mer counts were used to represent the antimicrobial resistance regions within a bacterial genome, and were then used to train the algorithm, which resulted in an outstanding performance with an AUC of 0.991 and an accuracy of 99.5%, similar to the model developed by Drouin et al. [15], which demonstrated a high accuracy of 98.7% for *Staphylococcus aureus*.

#### *Enterococcus faecium*

Drouin et al. [15] developed a set covering machine model to predict the antibiotic susceptibility status of 12 pathogens, including *Staphylococcus aureus, Enterococcus faecium, Escherichia coli, Klebsiella pneumoniae* and *Pseudomonas aeruginosa,* using 56 different antibiotics. The DNA k-mer counts were also extracted from the Pathosystems Resource Integration Center database. The model had a very high accuracy of 100% for *Enterococcus faecium*. The model was also highly interpretable and had a short computing time as a result of the implementation of the sample compression theory and the addition of some comprehensive tutorials that could guide users with no prior knowledge of ML to interpret the model.

#### *Escherichia coli*

Moradigaravand et al. [16] trained a gradient-boosting decision tree as an ML model to predict the resistance of *Escherichia coli* to 11 common antibiotics. Data including gene contents, isolation year, population structure and information on polymorphism were collected from 1936 whole genome sequencing samples of *Escherichia coli* strains for model training. The performance of the ML model was compared with that of a rule-based method developed in the same study, and the result demonstrated that the ML model, with an average accuracy of 91.0%, outperformed the rule-based method as well as the model developed by Drouin et al. [15], which achieved an accuracy of 81.8% for *Escherichia coli*.

#### *Klebsiella pneumoniae*

Drouin et al. [15] developed a model based on a binary phenotype classification, i.e., susceptible or resistant, and achieved an accuracy of 95.0% for *Klebsiella pneumoniae*. In another study by Nguyen et al. [17], the minimal inhibitory concentration (MIC) of *Klebsiella pneumoniae* was predicted by using a gradient-boosting tree model to determine the level of antibiotic resistance. The model was trained on 1668 isolates and achieved an overall accuracy of 92.0%.

#### *Pseudomonas aeruginosa*

Using genomic sequences and transcriptional data from 414 *Pseudomonas aeruginosa* samples, Khaledi et al. [18] developed a support vector machine classifier for predicting the susceptibility of *Pseudomonas aeruginosa* to four commonly used anti-pseudomonas antibiotics, including ceftazidime, meropenem, ciprofloxacin and tobramycin. The model achieved a high sensitivity and predictive value of 0.8–0.9 or > 0.9, which was comparable to the model of Drouin et al. [15], with an accuracy of 93.9% for *Pseudomonas aeruginosa*. Although more and more studies have developed predictive models for different species, there is still little effort in developing software that provides easy accessibility to the public who may not have high-standard computing hardware. Considering this, Aun et al. [19] developed a simple software called ‘PhenotypeSeeker’ that used MIC values as well as binary phenotypes to determine *Pseudomonas aeruginosa* resistance to ciprofloxacin using two regression models. This method achieved an accuracy of 88.0%. K-mers of 200 genomes were collected to develop the model. With assembled genomes, the model could be built in less than 5 h per phenotype and could generate a phenotype prediction in just a second.

### Prognosis

Shohat et al. [20] developed a random forest analysis model to predict DAIR failure using the patients’ demographics, medical comorbidities, microbiology, operative findings and laboratory findings. The cohort of total knee and hip arthroplasty patients contained 609 TKA cases. Significant predictors for the knee cohort and early acute PJI patients were positive blood cultures and high C-reactive protein, whereas days of symptoms and immunosuppression were more significant in late acute PJI patients. The model performance was acceptable with an AUC of 0.74.

Another model targeting revision TKA (rTKA) failure rate was developed by Klemt et al. [21], who used an artificial neural network to predict recurrent PJI following rTKA. The model was established by using 618 PJI cases with rTKA as the treatment. The model achieved an AUC of 0.84, a Brier score of 0.053 (close to zero indicating good accuracy of the probabilistic prediction), and a calibration intercept of 0.06 (indicating a slight underestimation of the risk prediction). Irrigation and debridement with or without modular component exchange during rTKA, more than four prior open surgeries, metastatic disease and drug abuse were identified as statistically significant variables for the prediction.

## Discussion

### Prediction

In clinical practice, TKA patients at high risk of developing PJI are identified based on the presence or absence of risk factors. However, there is currently no universal guideline on this matter, and clinicians can only predict the risk of PJI based on their experiences. Whenever a patient’s condition is complex or the dataset is incomplete, the difficulty of prediction may increase, requiring more time for an accurate prediction. The ML model presented by Yeo et al. [7] may be a promising alternative to the manual risk prediction method.

Applying ML models in PJI prediction following TKA has several benefits: First, the early preoperative risk prediction of PJI may assist in preoperative treatment decisions by allowing patients to weigh potential risks against benefits [7]. Second, the ML model may assist in the preoperative optimization of the patient’s condition [22] by identifying and correcting modifiable high-risk variables prior to surgery. Third, the ML model may be able to identify relationships between variables even in a complex and incomplete dataset. It may generate a prediction faster than a manual prediction, facilitating preoperative decision-making.

### Diagnosis

To date, there is no universal definition of PJI. Nonetheless, a widely accepted definition has been introduced by the Musculoskeletal Infection Society, which was endorsed at the 2013 ICM. The strength of the definition was further enhanced in a new version formulated by the Musculoskeletal Infection Society in 2018 [23]. Yet, having a fixed list of criteria and being non-specific to individual cases, the definition may not be able to provide personalized diagnostic approaches. The ML model suggested by Kuo et al. [8] may be an alternative for PJI diagnosis as it could provide a patient-specific explanation and aid in individualized decisions for PJI diagnosis. The decision diagram and the if-then rule used may also provide a more comprehensive explanation of the decision compared to the importance level presented by most other studies.

Currently, there is also no gold standard for pathological PJI diagnosis. The 2018 ICM pathological criteria [23] suggested that more than five neutrophils per high-power field observed in five high-power fields are needed for pathological diagnosis of PJI, whereas the European Bone and Joint Infection Society definition in 2021 [24] suggested that at least five neutrophils per high-power field observed in at least one high-power field are enough to suggest a possible PJI. The model suggested by Tao et al. [13] may be an alternative approach with the following advantages: First, the ML model may avoid the controversy of neutrophil number and positive high-power field number, as the diagnosis does not rely on the neutrophil count alone. It is also accompanied by several infection indicators such as tissue edema, capillary hyperplasia, neutrophil infiltration and proliferation. This could be a more comprehensive approach than the current pathological diagnostic criteria. Second, the ML method may be more accurate as it covers the entire pathological section and does not rely on the neutrophil count alone. In contrast, manual diagnostic methods only select suspected sections for recognition, which often omit pathological sections that may be infected, and only rely on the neutrophil count, which may be confused by the diverse morphology of neutrophils that may be similar to other inflammatory cells. Third, the ML method may shorten the time spent on pathological diagnosis. This is because ML can process multiple images at the same time. It may also be more powerful at recognizing pathological features than the manual method, enabling early diagnosis and thus early surgical intervention for PJI. Lastly, pathological diagnosis by ML could be more objective than the manual method which heavily depends on pathologists’ experience in recognizing pathological features.

### Antibiotic application

If a diagnosis of PJI is established, an antibiotic prescription will be urgent. However, it was anticipated that antibiotic resistance would result in a decrease in the effectiveness of antibiotics [25]. In view of this, it is important to know the antimicrobial susceptibility status before prescribing antibiotics for PJI treatment. Currently, microbial resistance to antibiotics can be identified by several methods, each with its own downsides. First, culture-dependent antimicrobial susceptibility testing, although commonly used, usually takes 12–48 h and is time-consuming [26]. For slow-growing microorganisms, days to weeks may be needed. For non-culturable PJI pathogens, there may even be no results [9]. Second, another method is the polymerase chain reaction-based method, which is limited by the completeness of the database of known antimicrobial resistance marker genes [9]. An alternative approach may be the next-generation sequencing-based predictive antimicrobial susceptibility testing with an ML model [14–19]. Some of the ML models included in the analysis used the binary phenotype classification, i.e., susceptible or resistant [14–16, 18]. Another predicted outcome used was the MIC, which further assessed the degree of susceptibility status [17, 19, 27].

Applying ML in the antibiotic prescription for PJI has several advantages: First, no prior knowledge of the resistance mechanism of the microbial strains is required to use the models [15–18], allowing for a more extensive application of the models. Second, the prediction could be generated in a short time, allowing for an early prescription of antibiotics. Third, ML models may identify the antibiotic susceptibility status of non-culturable PJI pathogens, which are the causative pathogens in 5%–42% of PJI [28], hence allowing for a more effective treatment prescription.

### Prognosis

Treatment options for PJI include DAIR, one- or two-stage rTKA, arthrodesis and amputation [29]. Among them, DAIR and rTKA are the two most common choices, but the success rates of these treatments vary greatly. For acute postoperative PJI, DAIR has a failure rate of 0–69%, whereas for late chronic PJI, the failure rate is 38%–72% [30–41]. One-stage rTKA has a failure rate of 27% and the rate of two-stage is less than 10% [42–50]. To improve the prognosis after treatment, an early and correct decision on the treatment option, and preoperative optimization of the patient’s condition are important.

Currently, there is a guideline for DAIR recommendation. However, the Infectious Diseases Society of America guideline published in 2013 may have multiple weaknesses, such as no separate guidance between early and late acute PJI, and little consideration of patient- and implant-related variables [51]. Alternatively, Shohat et al. [20] and Klemt et al. [21] demonstrated that ML models could provide more comprehensive treatment guidance than traditional guidelines. One special feature of Shohat’s [20] model is the separate analysis of the DAIR failure rate in early and late acute PJI patients given the reported difference in their failure rates [30–41]. The ML model developed by Klemt et al. [21] also out-performed a prior model with a conventional statistical approach [52].

Adapting ML models in prognostic prediction has several advantages: First, the prediction of treatment failure risk may assist clinicians and patients in making early preoperative treatment decisions, to better allocate resources, with revision surgery reserved for patients at high risk and DAIR for patients at low risk. Second, an individualized prediction may lead to more patient-specific guidance than conventional guidelines due to the involvement of more patient-specific variables. Third, ML models allow for preoperative optimization of patients’ conditions, thereby reducing the failure rate by correcting modifiable risk factors prior to surgery. Lastly, the risk prediction may allow for early preparation for prescriptions, lengthened hospital stays, and subsequent treatment planning for possible treatment failure in high-risk patients.

### Limitations

While ML could be an effective tool for preventing PJI, it does have several practical limitations that restrict its use. First, ML algorithms have ‘black box problems’, meaning that their decision-making processes are not transparent, their results may not be interpretable, and their flaws may not be readily detectable [53]. It is therefore essential to evaluate and validate the algorithm extensively before putting it into clinical practice [54]. Second, ML algorithms are likely to overfit in imbalanced datasets. In the case of overfitting, an algorithm with high accuracy may not perform well when tested on an unseen dataset [5, 55]. Third, a large database with millions to trillions of data points may be required for training and testing [5], and a separate set of local training data has to be available to adapt the algorithm to a new population [56]. Hence, hospitals with small data sizes may need data sharing, which presents a problem of data protection and privacy infringement [57, 58]. With the emergence of massive databases, such as the National Inpatient Sample datasets and the American College of Surgeons-National Surgical Quality Improvement Program database, more abundant datasets will be available to facilitate future research on ML applications.

Although ML application is gaining more attention, there are research gaps to be bridged. The studies reviewed above were not of prospective nature and were not externally validated. There is also a paucity of research in PJI-related areas. Future studies on the AI-based prediction of the risk of PJI with a longer follow-up time are needed and should cover more commonly used antibiotics and pathogens in investigating the susceptibility of the microbes. In addition, most of the current models did not assess the impact of the severity of disease factors on the outcome [7, 8, 13–21]. For example, only the presence or absence of hypertension was examined for its association with PJI risk following primary TKA without taking into account the severity of hypertension. This provides a new direction for future research work.

## Conclusion

Machine learning may be a favorable alternative to manual methods in the prevention of PJI after TKA. It aids in preoperative patient optimization, preoperative surgical planning, early diagnosis of infection, early application of suitable antibiotics, and the prediction of clinical outcomes. Although ML applications are potentially beneficial to the prevention of PJI, some current limitations need to be overcome in order to ensure that ML is a non-inferior or even superior option to manual approaches and, therefore, worth application in clinical settings.

## Data Availability

All data analyzed during this study are included in this published article.

## Abbreviations

AUC: Area under the receiver operating characteristic curve
DAIR: Debridement, antibiotics and implant retention
ICM: International Consensus Meeting
MIC: Minimum inhibitory concentration
ML: Machine learning
PJI: Periprosthetic joint infection
rTKA: Revision total knee arthroplasty
TKA: Total knee arthroplasty
